# The impact of expanded access to direct acting antivirals for Hepatitis C virus on patient outcomes in Canada

**DOI:** 10.1371/journal.pone.0284914

**Published:** 2023-08-08

**Authors:** Cherry Chu, Tara Gomes, Tony Antoniou, William W. L. Wong, Naveed Janjua, Jason Robert Guertin, Kevin L. Schwartz, Jordan Feld, Jeff Kwong, Mina Tadrous

**Affiliations:** 1 Institute for Health System Solutions and Virtual Care, Women’s College Hospital, Toronto, ON, Canada; 2 Unity Health Toronto, Toronto, ON, Canada; 3 Leslie Dan Faculty of Pharmacy, University of Toronto, Toronto, ON, Canada; 4 Institute of Health Policy, Management and Evaluation, University of Toronto, Toronto, ON, Canada; 5 Institute for Clinical Evaluative Sciences, Toronto, ON, Canada; 6 Department of Family and Community Medicine, University of Toronto, Toronto, ON, Canada; 7 School of Pharmacy, University of Waterloo, Kitchener, ON, Canada; 8 BC Centre for Disease Control, Vancouver, BC, Canada; 9 School of Population and Public Health, University of British Columbia, Vancouver, BC, Canada; 10 Département de médecine sociale et préventive, Faculté de médecine, Université Laval, Quebec City, QC, Canada; 11 Centre de recherche du CHU de Québec-Université Laval, Quebec City, QC, Canada; 12 Centre de Recherche en Organogénèse expérimentale de l’Université Laval/LOEX, Quebec City, QC, Canada; 13 Public Health Ontario, Toronto, ON, Canada; 14 St. Joseph’s Health Centre, Unity Health Toronto, Toronto, ON, Canada; 15 Dalla Lana School of Public Health, University of Toronto, Toronto, ON, Canada; 16 Toronto Centre for Liver Disease, University Health Network, Toronto, ON, Canada; Centers for Disease Control and Prevention, UNITED STATES

## Abstract

**Background:**

Hepatitis C virus (HCV) has high global prevalence and can lead to liver complications and death. Access to direct-acting antivirals (DAAs) in Canada increased following several policy changes, however the real-world impact of expanded DAA access and increased use of these drugs is unknown.

**Objective:**

We aimed to determine the early change in rates of HCV-related hospitalizations overall and HCV-related hospitalizations with hepatocellular carcinoma (HCC) after expanded DAA access.

**Methods:**

We conducted a population-based time series analysis using national administrative health databases in Canada. Rates of HCV-related hospitalizations and HCV-related hospitalizations with HCC were enumerated monthly between April 2006 and March 2020. We used Autoregressive Integrated Moving Average (ARIMA) models with ramp functions in October 2014 and January 2017 to evaluate the impact of policies to expand DAA access on hospitalization outcomes.

**Results:**

Rates of HCV-related hospitalizations in Canada increased between 2006 and 2014, and gradually declined thereafter. The decrease after October 2014, or the first policy change, was significant (p = 0.0355), but no further change was found after the second policy change in 2017 (p = 0.2567). HCV-related hospitalizations with HCC increased until end of 2013, followed by a plateau, before declining in 2016. No significant shifts were found after the first policy change in 2014 (p = 0.1291) nor the second policy change in 2017 (p = 0.6324). Subgroup analyses revealed that those aged 50–64 and males had observable declines in rates of HCV-related hospitalizations in the year prior to the first policy change.

**Conclusions:**

Expanding DAA access was associated with a drop in HCV-related hospitalizations in the overall Canadian population coinciding with the 2014 policy change. In light of the time required for HCV-related complications to manifest, continued ongoing research examining the real-world effectiveness of DAAs is required.

## Introduction

Hepatitis C virus (HCV) is a life-threatening condition affecting 58 million people worldwide [1]. HCV accounts for approximately 40% of all chronic liver disease cases [2] and is the top cause of liver-related deaths, cirrhosis, and hepatocellular carcinoma [3]. Early treatment for HCV with interferon-based therapy and ribavirin demonstrated low cure rates [4]. Direct-acting antivirals, first introduced in 2011, have cure rates exceeding 95% and reduce mortality in patients with HCV [5, 6]. However, DAA treatment is costly and has led to the rationing of care to certain subgroups such as those with advanced liver disease [7].

In Canada, many provinces and territories initially limited DAA access to patients with moderate fibrosis with access to specialist care, with some regions further restricting access to patients who did not have active substance use or HIV [8]. Subsequent policy changes expanded access to DAAs. Specifically, in October 2014, the first DAA was approved for use by Health Canada [9]. In 2017 DAAs were transferred to the general formulary with restrictions on use. In 2018, all disease-based criteria for treatment were removed and newer agents were included in the general formulary [10]. DAA use increased as restrictions governing access were removed, with public payers comprising the vast majority of payments [11]. In Ontario alone, the number of DAA beneficiaries grew from 13 per quarter in 2012 to a peak of 2539 in 2017 [10]. Despite greater uptake, there is limited evidence on the real-world patient outcomes of this policy change in the overall Canadian population. An earlier study found a modest decrease in HCV and liver disease-related hospitalizations from 2012 to 2016 [12]. However, the impact of policies intended to increase DAA access on hepatitis C-related hospitalizations is unknown.

Accordingly, the goal of this study was to assess changes in overall HCV-related hospitalizations, and HCV-related hospitalizations with hepatocellular carcinoma (HCC) after expanded DAA access in Canada.

## Methods

This study received exemption from the Women’s College Hospital Research Ethics Board. Individual consent was waived due to the use of routinely-collected administrative data. We conducted a population-based study using national administrative databases in Canada from the Canadian Institute for Health Information. We specifically used the Discharge Abstract Database (DAD), which captures hospital discharges from acute inpatient care facilities in all provinces and territories except Quebec, and the Hospital Morbidity Database (HMDB), which records all hospital discharges in Quebec.

All acute inpatient records for Canadians with any diagnosis of hepatitis C virus (HCV), chronic liver disease (CLD), or hepatocellular carcinoma (HCC), with or without a recorded liver transplant, between April 2006 and March 2020 were identified. We further restricted these records to only those with any diagnosis code of B18.2 and/or B17.1 indicating acute or chronic HCV. A list of codes used to apply these inclusion criteria are available in the **S1 Appendix** [13].

The following outcomes were measured monthly between April 2006 and March 2020: 1) Rate of HCV-related hospitalizations overall and 2) Rate of HCV-related hospitalizations with HCC. National population denominators were extracted from census data [14]. Since the census data provided quarterly estimates, we extrapolated in between quarters to estimate monthly numbers. We conducted an interrupted time series analysis using interventional Autoregressive Integrated Moving Average (ARIMA) modelling for each outcome, a common statistical method for analyzing the impact of a program intervention or policy change to a time series. Ramp functions in October 2014 and January 2017 were incorporated into the models to identify any gradual changes to the rate of hospitalizations at the two timepoints during which policies were implemented to expand DAA access. Any seasonality or non-stationarity was removed using differencing where necessary. Stationarity was confirmed using the Augmented Dickey-Fuller test. Moving average or autoregressive terms were chosen using autocorrelation function and partial autocorrelation function plots. Model fit was determined using residual checks such as the Ljung Box test for white noise and comparison of various model fit statistics such as the Akaike Information Criterion and Schwarz-Bayesian Information Criterion.

We also report descriptive results from the following secondary analyses: 1) Analysis of yearly HCV-specific hospitalization rates stratified by age group; 2) Analysis of yearly HCV-specific hospitalization rates stratified by sex; 3) Analysis of monthly HCV-specific hospitalization rates with HCV prevalence estimates as denominators; 4) Analysis of monthly total liver-related hospitalization rates. For the age and sex group denominators, since we only had access to the population as of July 1st of each year, we assumed that to be the population for that fiscal year. For HCV prevalence denominators, we had access to yearly estimates from a previous study which we extrapolated to estimate monthly data [15].

P-values less than 0.05 were considered statistically significant. All analyses were conducted using SAS Version 9.4.

## Results

**Fig 1:**
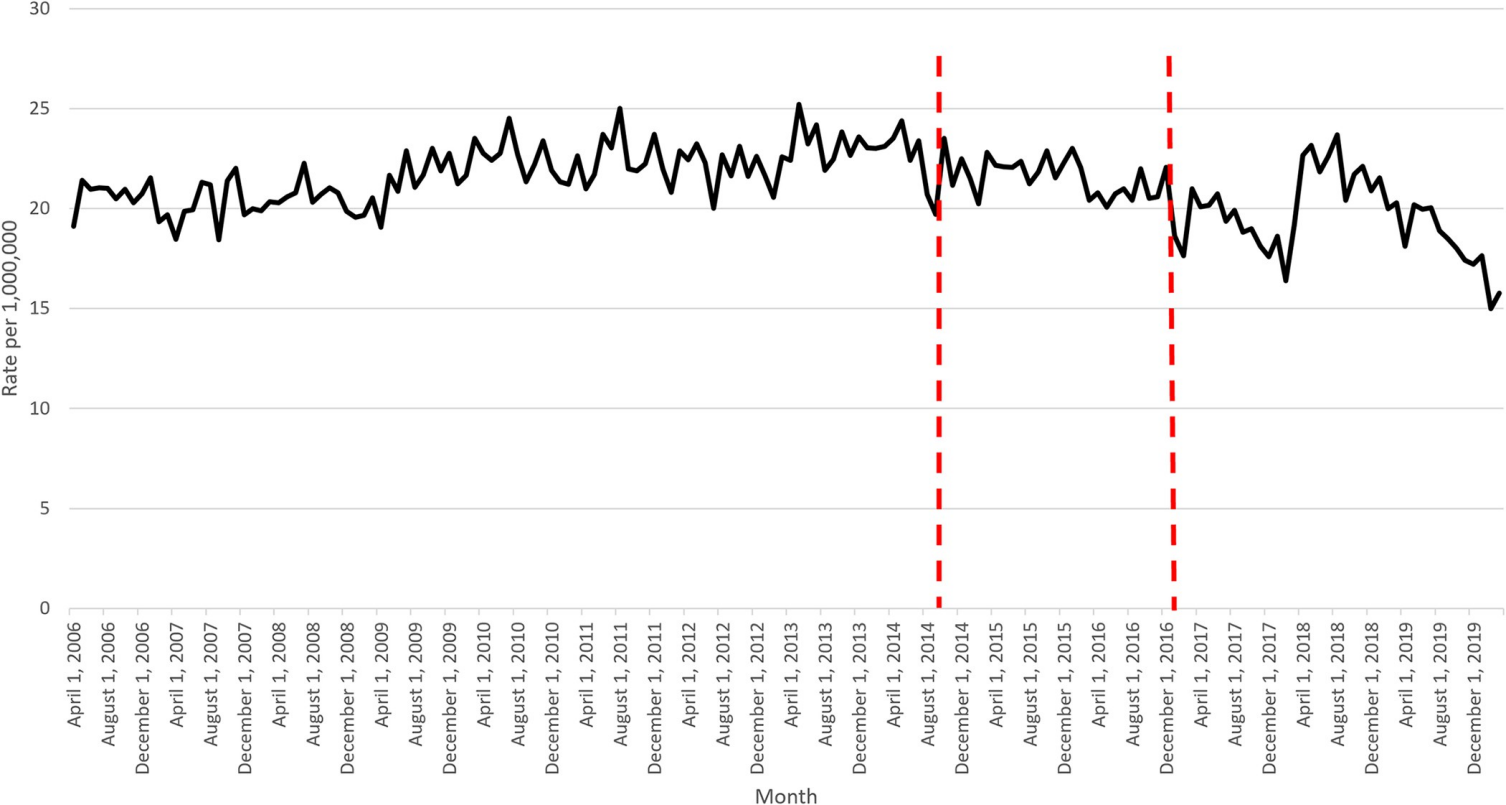
Monthly rate of HCV-specific hospitalizations per 1,000,000 of Canadian population.

Rates of HCV-related hospitalizations in Canada increased between 2006 and 2014, and gradually declined thereafter (**Fig 1**). This was followed by a sudden spike in early 2018, and then another decline. Slopes are reported in **Table 1**. The average rate of admissions per month prior to October 2014 (first policy change) was 21.7 per 1,000,000 (slope of +0.000982553) versus 21.6 between October 2014 to December 2016 (slope of -0.001753523). Subsequently, the average rate of admissions per month from January 2017 (second policy change) to March 2020 was 19.6 per 1,000,000 (slope of -0.00153088). The decrease after October 2014 was significant (p = 0.0355), but no further change was found after January 2017 (p = 0.2567).

**Table 1:** Slopes.

Time period	HCV-specific hospitalizations (slope)	HCV-specific hospitalizations with HCC (slope)
April 2006 to September 2014	+0.000982553	+0.000373055
October 2014 to December 2016	-0.001753523	-0.000181163
January 2017 to March 2020	-0.00153088	-0.000502179

**Fig 2:**
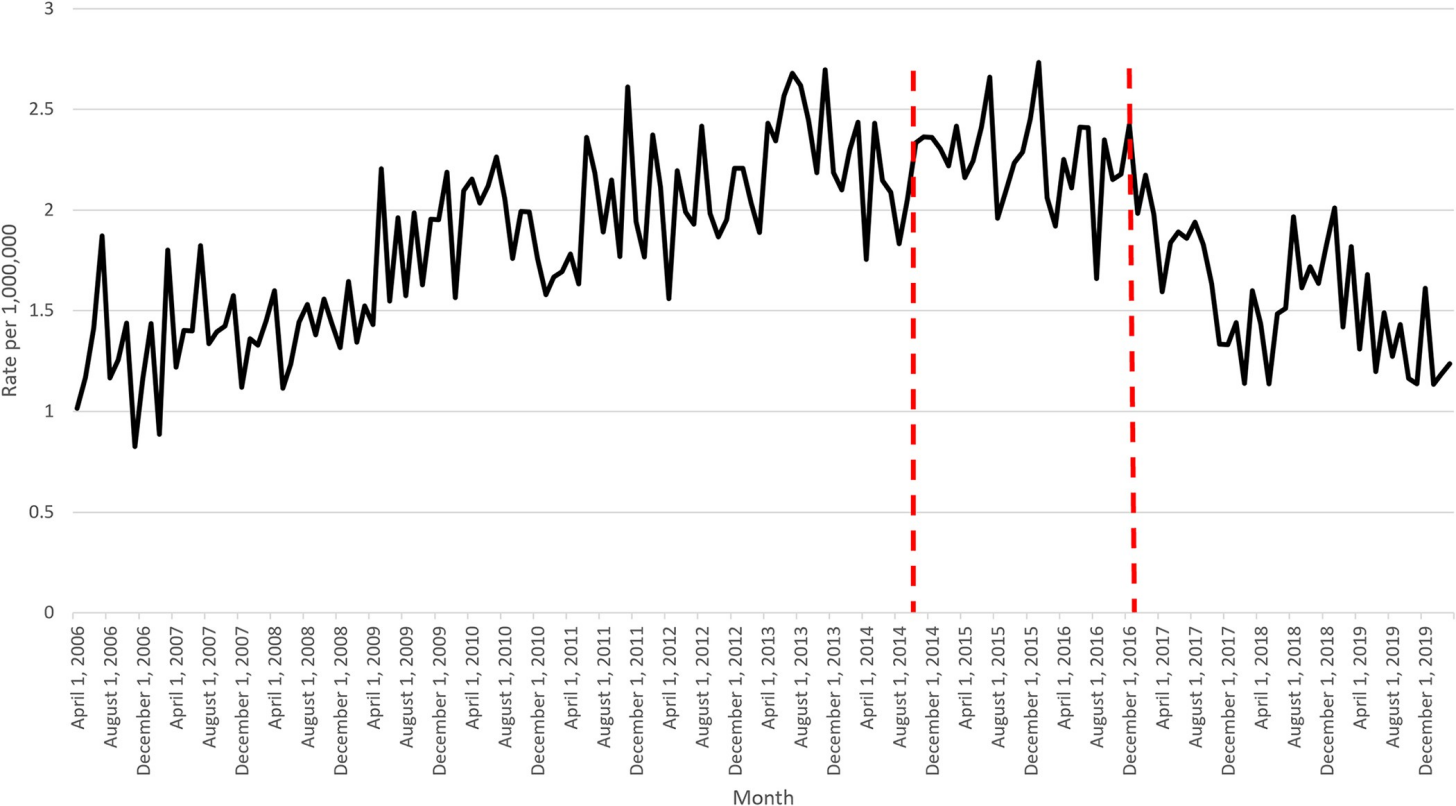
Monthly rate of HCV-specific hospitalizations with HCC per 1,000,000 of Canadian population.

HCV-related hospitalizations with HCC increased until end of 2013, followed by a plateau, before declining in 2016 (**Fig 2**). This was followed by a small uptick after mid-2018 and then a subsequent decline. The average rate of admissions per month prior to October 2014 (first policy change) was 1.8 per 1,000,000 (slope of +0.000373055) versus 2.3 between October 2014 to December 2016 (slope of -0.000181163). Subsequently, the average rate of admissions per month from January 2017 (second policy change) to March 2020 decreased to 1.6 per 1,000,000 (slope of -0.000502179). No significant shifts were found after the first policy change in 2014 (p = 0.1291) nor the second policy change in 2017 (p = 0.6324).

Descriptive results from the secondary analyses are shown in the **Supplemental Figures**. Notably, HCV hospitalization rates were highest among those aged 50–64 (average 602 admissions per 1,000,000 per year), however the rate peaked in 2013 and gradually decreased thereafter (**S1 Fig**). HCV hospitalization rates were higher among males (average 329 admissions per 1,000,000 per year) than females, with the rate similarly reaching a peak in 2013 and decreasing thereafter (**S3 Fig**). Across the remaining age and sex groups, the hospitalization rates remained relatively stable. When compared to HCV-specific hospitalizations out of the entire Canadian population, rates were much higher for HCV-specific hospitalizations using the HCV patient population as the denominator (average 5678 admissions per 1,000,000 per year), however the trend remained similar (**S5 Fig**). In contrast, total liver-related hospitalization rates continuously increased throughout the study period (average 175 admissions per 1,000,000 per month), with no visible changes to trajectory after the policy changes (**S7 Fig**).

## Discussion

In our population-based study, we found that the expansion of DAA access in 2014 led to a significant decline in HCV-related hospitalization rates overall but not in hospitalizations with hepatocellular carcinoma. No further change in trajectory was observed following full listing of DAAs in 2017. Stratifications by age and sex revealed that those aged 50–64 and males began to show declining rates in the year leading up to the first policy change. For the remaining subgroups, however, there did not appear to be any observable fluctuations coinciding with the policy changes.

Many studies report on the efficacy of DAA treatment among those who have been treated [16–19]–however limited studies have evaluated the population-level impact of DAA access on HCV and liver-related morbidity and mortality. Our findings are somewhat consistent with those from the previous iteration of this study using earlier data, which found that HCV and liver disease-related hospitalizations decreased during the time of better DAA access [12]. However, in our study we did not consistently identify significant population-wide reductions across both hospitalization outcomes due to improved DAA availability. It is possible that we are still underutilizing treatment in the population and therefore not maximizing the full potential of DAAs, as another Canadian study found that socio-economically marginalized populations were less likely to receive DAA treatment [20]. Furthermore, two population-based studies from the US and Australia, respectively, cited that although inpatient mortality decreased during the DAA era among HCV patients, there is still substantial disease burden among those with substance use disorder [21, 22]. This may be due to disparities in access to DAA treatment within this subgroup. DAA has shown to be an effective treatment method for HCV among substance users, as one longitudinal study cited that fewer persons who inject drugs had detectable HCV RNA and users experienced sharp decreases in liver morbidity and mortality following HCV treatment [23]. As HCV is commonly associated with substance use [24], efforts should be made to reduce barriers to treatment in this population.

The absence of consistent reductions in our study outcomes also suggests that a longer follow-up period is likely needed to detect any sustained declines in HCV-related hospitalizations given that the policy changes in Canada are recent and HCV necessitates decades of progression from infection to the stage of advanced liver disease requiring hospitalization. This is especially the case for those who received DAA because of the more liberal policy implemented in 2017 (second policy change). HCV incidence and prevalence will likely decrease as DAA treatment becomes more universal which will influence the outcomes of this study, however more lead time will be required to detect any major signals.

We found that, when stratifying by age group, patients aged 50–64 had the highest rates of HCV-related hospitalizations. This age group also had the most obvious drop in hospitalization rates compared to other age groups, and the reduction appeared to occur not during the year of the policy change, but the year prior. This may be due to patients receiving DAA treatment through other mechanisms, as DAA dispensing increased slightly in 2013 [10]. Our previous iteration of this study also reported the largest reductions in hospitalizations associated with HCV and chronic liver disease among those aged 55 and above, while short-term reductions in hospitalizations could not be found among the younger cohort [12]. Studies have shown that HCV prevalence is the highest in those aged 50 and above, potentially due to historical reasons such as higher incidence of HCV infections in the 1970s or 1980s when these individuals were young adults, and changes in injection practices and precautions against blood-borne pathogen transmission. These individuals were likely infected decades ago, with serious illness and hospitalizations manifesting once they reached this age bracket [3]. Several studies have reported on the high cost-effectiveness of antiviral treatment for Hepatitis C [25, 26], and given that much of the disease burden lies in the 50+ age group, efforts should be placed into targeting this group for intervention. Conversely, we see that younger patients in the age <30 and 30–49 groups have low rates of hospitalizations throughout, and therefore any major changes are unlikely to be captured until the future.

We note that, in contrast to the trajectory for HCV-specific hospitalizations, the trend in total liver disease-related hospitalizations was continuously increasing throughout, likely due to the burden of other causes of liver disease besides hepatitis, such as nonalcoholic fatty liver disease or alcohol abuse, which remain unaffected by improved DAA access.

Limitations of this study include the use of administrative databases which, while able to capture population-level outcomes, inherently lack patients’ clinical details and therefore we were unable to identify a confirmatory diagnosis. We were also limited to annual data when stratifying outcomes by age and sex as opposed to monthly data in the main findings, therefore direct comparisons are difficult to make. Furthermore, this study captures many years of data before the policy changes but only several years after the changes. Due to the time required for clinical changes to be manifested in the population after increased drug access, the results from this study only represent the early impact on outcomes. Interestingly, an increase in hospitalization rates in 2018 was seen across all trajectories that we assessed. The reason for this sudden spike remains unknown but may be attributed to both the changes in data coding and an increase in HCV testing that was driven by the listing of newer agents on the formulary.

Two policy changes were implemented in Canada to improve DAA access, however our study only found a significant reduction in HCV-related hospitalizations after the first policy change which likely impacted those at highest risk first. Despite the absence of significant changes in some instances, it has been well established that the introduction of DAA has significantly transformed the treatment and prognosis of patients with HCV. In some jurisdictions, HCV-related mortality reportedly improved after DAA treatment became free and widely accessible [27]. Future research should aim to evaluate outcomes at the patient-level to allow for more clinical granularity, and to continue to monitor longer term outcomes in the HCV patient population in Canada.

## Supporting information

S1 Checklist STROBE statement—Checklist of items that should be included in reports of observational studies. (DOCX)

S1 Appendix Codes for outcomes. (DOCX)

S1 Fig Yearly rate of HCV-specific hospitalizations per 1,000,000 of Canadian population, by age group. (PPTX)

S2 Fig Yearly rate of HCV-specific hospitalizations with HCC per 1,000,000 of Canadian population, by age group. (PPTX)

S3 Fig Yearly rate of HCV-specific hospitalizations per 1,000,000 of Canadian population, by sex. (PPTX)

S4 Fig Yearly rate of HCV-specific hospitalizations with HCC per 1,000,000 of Canadian population, by sex. (PPTX)

S5 Fig Monthly rate of HCV-specific hospitalizations per 1,000,000 of HCV patient population in Ontario. (PPTX)

S6 Fig Monthly rate of HCV-specific hospitalizations with HCC per 1,000,000 of HCV patient population in Ontario. (PPTX)

S7 Fig Monthly rate of total liver-related hospitalizations per 1,000,000 of Canadian population. (PPTX)

S8 Fig Monthly rate of total liver-related hospitalizations with HCC per 1,000,000 of Canadian population. (PPTX)

## Abbreviations

HCV: Hepatitis C Virus
DAA: Direct Acting Antivirals
HCC: Hepatocellular Carcinoma
ARIMA: Autoregressive Integrated Moving Average
DAD: Discharge Abstract Database
HMDB: Hospital Morbidity Database
CLD: Chronic Liver Disease
