# Supplementary material for: The impact of expanded access to direct acting antivirals for Hepatitis C virus on patient outcomes in Canada
Source: PLoS One. 2023 Aug 8;18(8):e0284914. doi: 10.1371/journal.pone.0284914 (PMC10409286; doi:10.1371/journal.pone.0284914)
Supplement: S1 Appendix — (DOCX) [file pone.0284914.s002.docx]

**S1 Appendix. Codes for Outcome**

**Codes used to identify acute inpatient records with any diagnosis of hepatitis C virus (HCV), chronic liver disease (CLD), or hepatocellular carcinoma (HCC)**

| **ICD-10-CA Code Description** | **ICD-10-CA Code** |
| --- | --- |
| Chronic viral hepatitis C | B18.2 |
| Acute hepatitis C | B17.1 |
| Malignant neoplasm of liver and intrahepatic bile duct | C22 |
| Alcoholic liver disease | K70 |
| Toxic liver disease | K71 |
| Hepatic failure, not elsewhere classified | K72 |
| Chronic hepatitis, not elsewhere classified | K73 |
| Fibrosis and cirrhosis of liver | K74 |
| Other inflammatory liver disease | K75 |
| Other disease of liver | K76 |
| Liver disorders in diseases classified elsewhere | K77 |
| Ascites | R18 |
