# Supplementary material for: The impact of expanded access to direct acting antivirals for Hepatitis C virus on patient outcomes in Canada
Source: PLoS One. 2023 Aug 8;18(8):e0284914. doi: 10.1371/journal.pone.0284914 (PMC10409286; doi:10.1371/journal.pone.0284914)
Supplement: S1 Fig — (PPTX) [file pone.0284914.s003.pptx]

## Slide 1
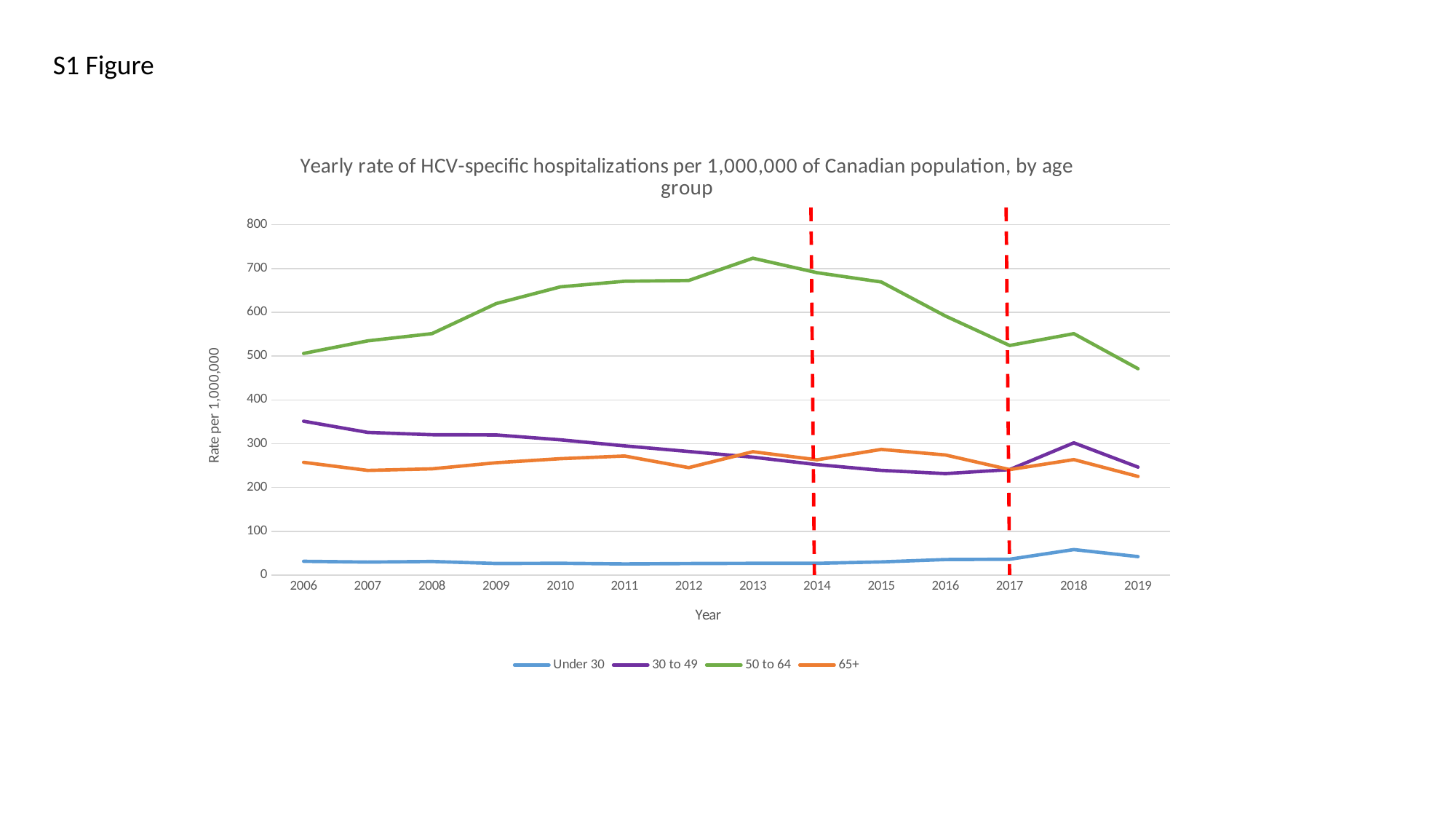

S1 Figure
### Chart: Yearly rate of HCV-specific hospitalizations per 1,000,000 of Canadian population, by age group
| Category | Under 30 | 30 to 49 | 50 to 64 | 65+ |
|---|---|---|---|---|
| 2006 | 31.636031764853037 | 351.33955287994206 | 506.0928708406213 | 257.4726099618746 |
| 2007 | 29.904303796606648 | 325.7397166938252 | 534.8332845747368 | 239.0656851629322 |
| 2008 | 31.19769398254525 | 320.46192180525253 | 551.2756453722967 | 242.67162521283313 |
| 2009 | 26.674433492707063 | 319.9218940847975 | 619.8382921392908 | 256.5681335096615 |
| 2010 | 27.090761381067892 | 308.8593815857647 | 657.9541234568832 | 265.81931988139087 |
| 2011 | 25.66390455252241 | 295.01953847526727 | 670.7188913938647 | 271.8337273610636 |
| 2012 | 26.520320938632533 | 282.18461410300347 | 672.5106797905629 | 245.39582152022422 |
| 2013 | 26.995165734136297 | 269.27979275681776 | 723.3399497212337 | 281.71208464514086 |
| 2014 | 27.07078309401266 | 252.29915104129802 | 690.2738369736396 | 263.24687504849044 |
| 2015 | 30.28640555746889 | 239.10752027428185 | 669.1331983375973 | 286.95071525349266 |
| 2016 | 35.6167524957502 | 231.80359845620043 | 591.4249347967622 | 274.27947592353144 |
| 2017 | 36.306612672772836 | 240.89497564107367 | 524.153921115222 | 240.91232421387275 |
| 2018 | 58.39135783446937 | 302.0351366866198 | 551.1469522603721 | 263.70644123437273 |
| 2019 | 42.348121675530656 | 246.53464921077435 | 471.2522002278791 | 225.3026889353198 |
