# Supplementary material for: The impact of expanded access to direct acting antivirals for Hepatitis C virus on patient outcomes in Canada
Source: PLoS One. 2023 Aug 8;18(8):e0284914. doi: 10.1371/journal.pone.0284914 (PMC10409286; doi:10.1371/journal.pone.0284914)
Supplement: S2 Fig — (PPTX) [file pone.0284914.s004.pptx]

## Slide 1
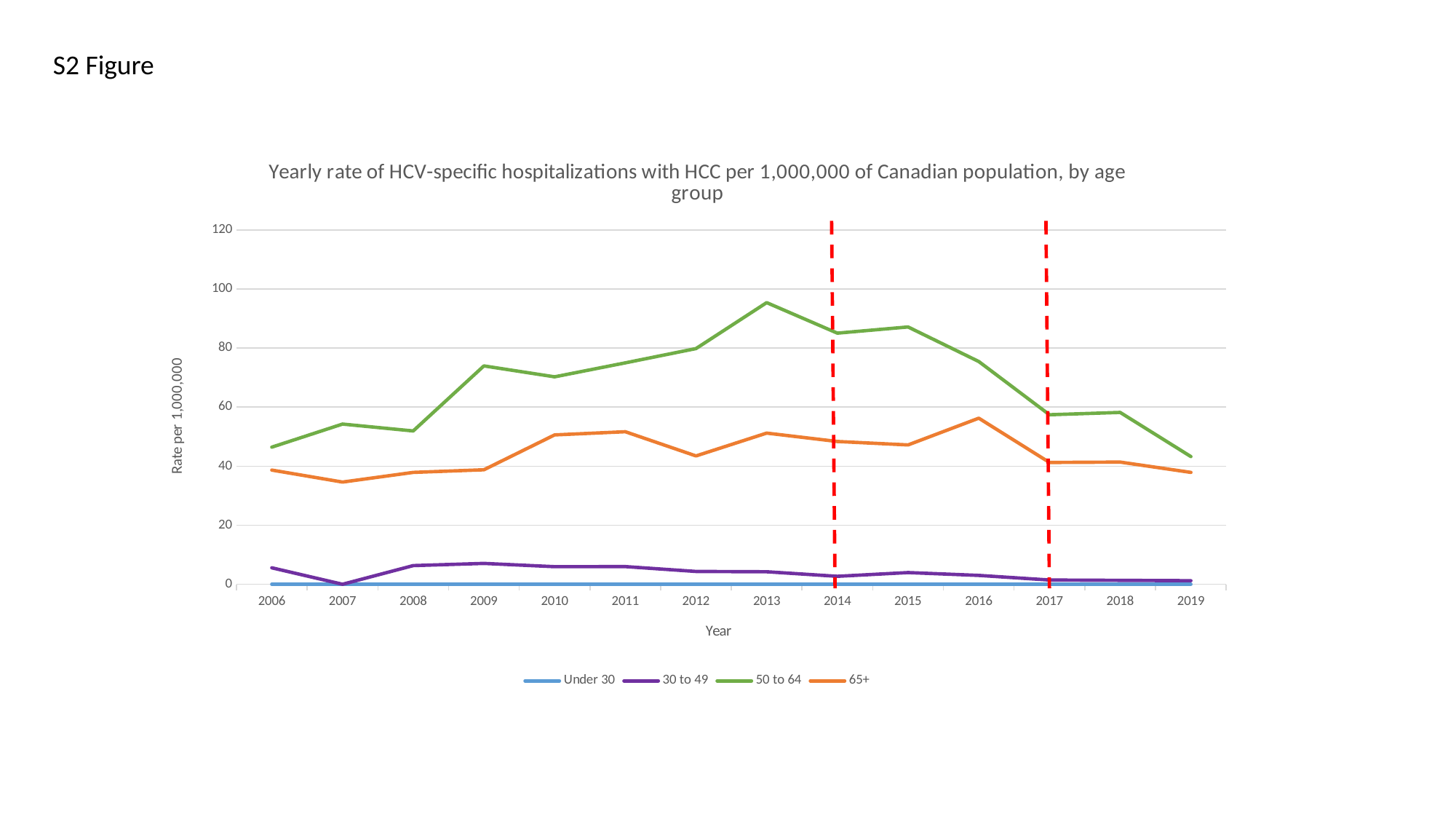

S2 Figure
### Chart: Yearly rate of HCV-specific hospitalizations with HCC per 1,000,000 of Canadian population, by age group
| Category | Under 30 | 30 to 49 | 50 to 64 | 65+ |
|---|---|---|---|---|
| 2006 | 0.0 | 5.565574714400004 | 46.42751775440963 | 38.667199517655625 |
| 2007 | 0.0 | 0.0 | 54.2469214872056 | 34.604588296999644 |
| 2008 | 0.0 | 6.319541714989077 | 51.922831886658464 | 37.8761520295892 |
| 2009 | 0.0 | 7.052591275351766 | 73.96816649203205 | 38.763632859139165 |
| 2010 | 0.0 | 5.949466666215328 | 70.27257209350107 | 50.58268968768832 |
| 2011 | 0.0 | 5.9766445098028305 | 74.99420979855738 | 51.66253467292672 |
| 2012 | 0.0 | 4.330198681887522 | 79.82692084738837 | 43.45348934429266 |
| 2013 | 0.0 | 4.234933449570206 | 95.39740063764489 | 51.186413257804105 |
| 2014 | 0.0 | 2.6906390184880014 | 85.0549202277877 | 48.35514908361579 |
| 2015 | 0.0 | 3.9470398655181196 | 87.15875417423034 | 47.18434416470342 |
| 2016 | 0.0 | 2.9983516303433597 | 75.4514055352292 | 56.24080386855663 |
| 2017 | 0.0 | 1.4369534124307761 | 57.42208146141649 | 41.238713143511376 |
| 2018 | 0.0 | 1.318487836442598 | 58.20523888357201 | 41.38114203140813 |
| 2019 | 0.0 | 1.1991956994443826 | 43.238869272683615 | 37.878730486771985 |
