# Supplementary material for: The impact of expanded access to direct acting antivirals for Hepatitis C virus on patient outcomes in Canada
Source: PLoS One. 2023 Aug 8;18(8):e0284914. doi: 10.1371/journal.pone.0284914 (PMC10409286; doi:10.1371/journal.pone.0284914)
Supplement: S3 Fig — (PPTX) [file pone.0284914.s005.pptx]

## Slide 1
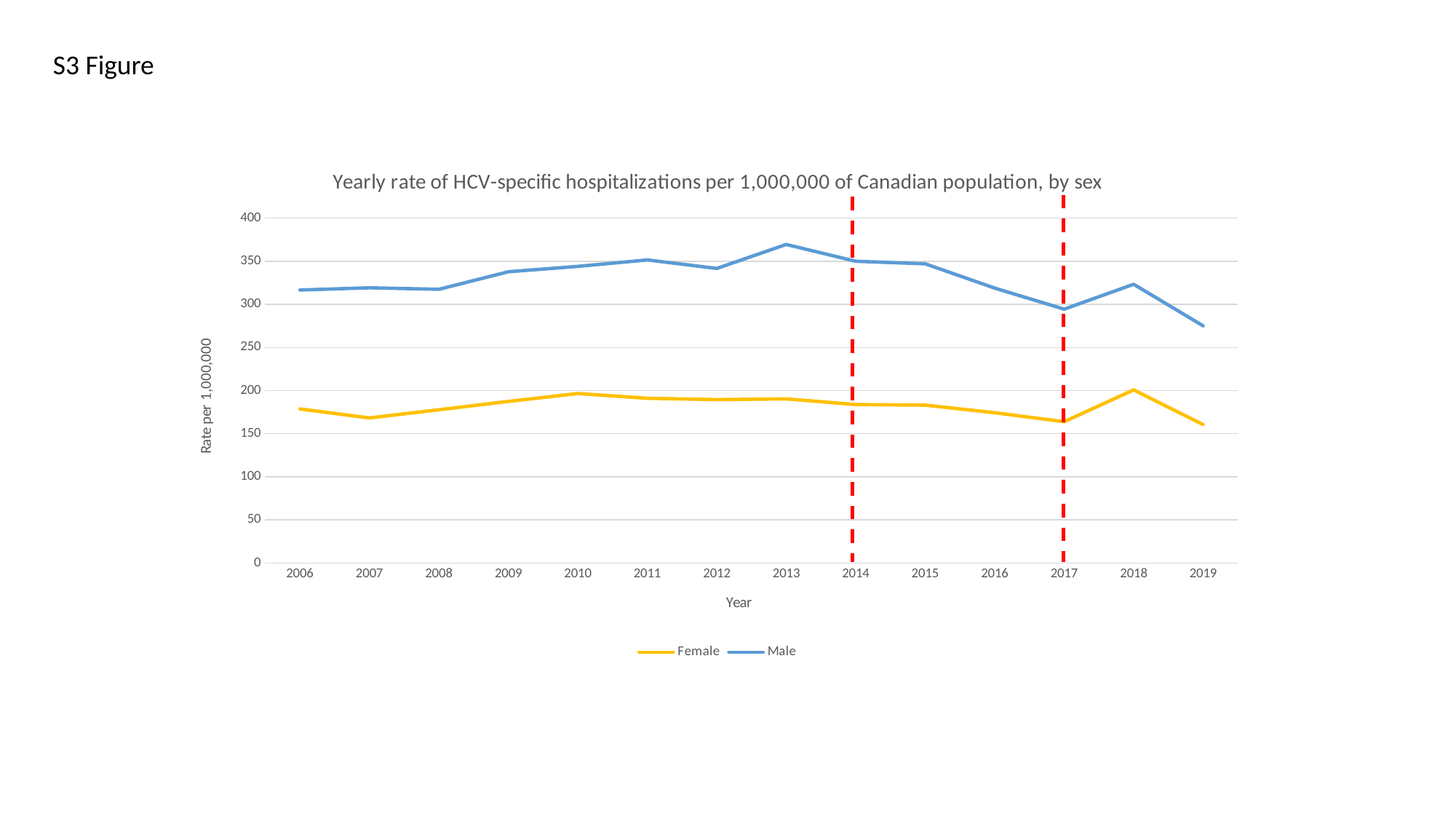

S3 Figure
### Chart: Yearly rate of HCV-specific hospitalizations per 1,000,000 of Canadian population, by sex
| Category | Female | Male |
|---|---|---|
| 2006 | 178.67562703121772 | 316.63526225445673 |
| 2007 | 168.23212150952253 | 319.2249368237714 |
| 2008 | 177.72674319469334 | 317.4665224571447 |
| 2009 | 187.4394137460993 | 337.8059464768712 |
| 2010 | 196.5953852482703 | 344.020708194762 |
| 2011 | 191.05559660140375 | 351.5231218873659 |
| 2012 | 189.5531857789179 | 341.6638097839035 |
| 2013 | 190.365352736649 | 369.45802191979675 |
| 2014 | 183.8064604218543 | 349.96621770924617 |
| 2015 | 183.15622024927367 | 347.0371512670413 |
| 2016 | 174.29789307321704 | 318.8681536836221 |
| 2017 | 163.8324806366956 | 294.4389416342618 |
| 2018 | 200.8167727828156 | 323.31349445656707 |
| 2019 | 160.5306550385456 | 275.0669401813708 |
