# Supplementary material for: The impact of expanded access to direct acting antivirals for Hepatitis C virus on patient outcomes in Canada
Source: PLoS One. 2023 Aug 8;18(8):e0284914. doi: 10.1371/journal.pone.0284914 (PMC10409286; doi:10.1371/journal.pone.0284914)
Supplement: S4 Fig — (PPTX) [file pone.0284914.s006.pptx]

## Slide 1
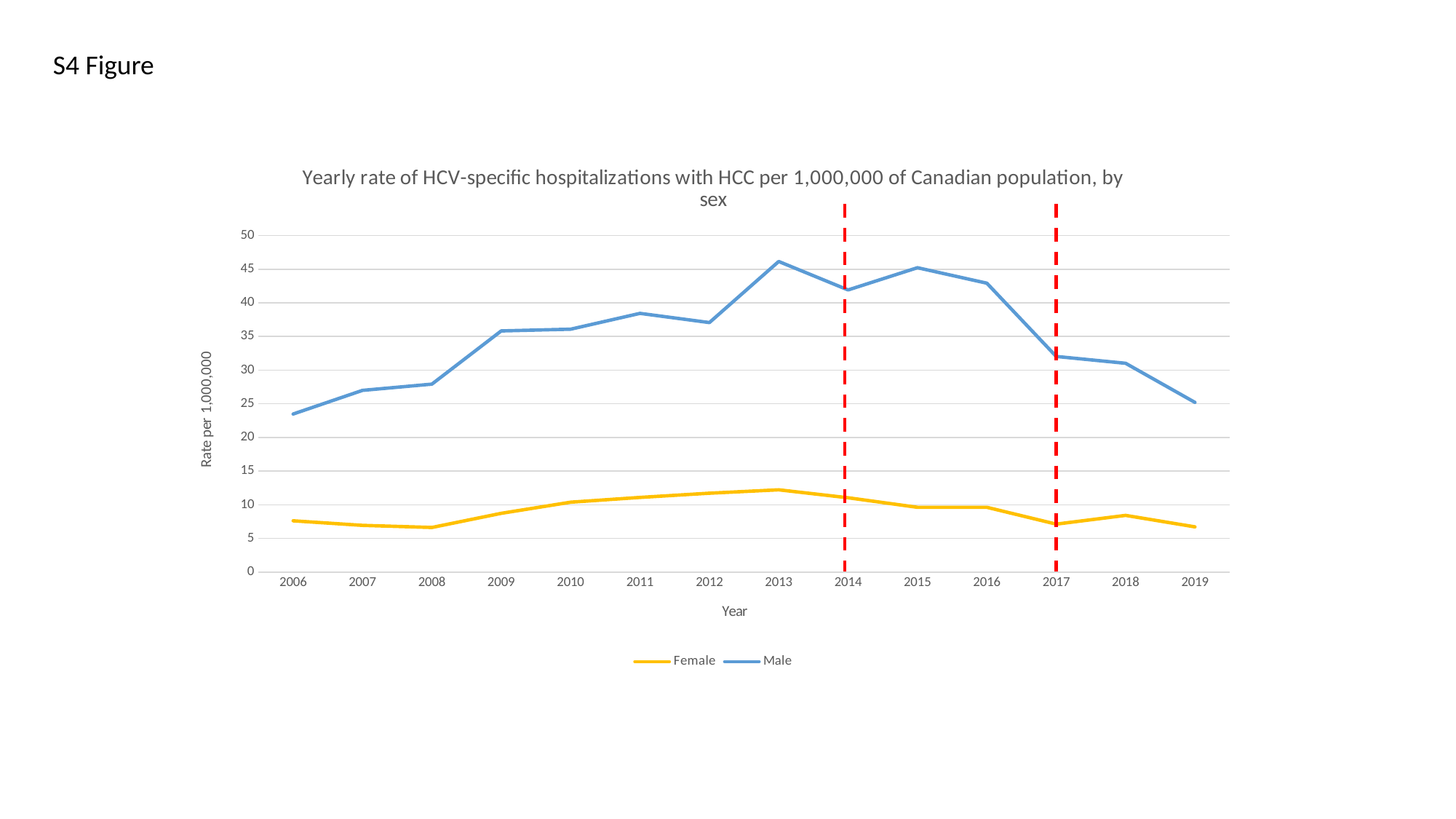

S4 Figure
### Chart: Yearly rate of HCV-specific hospitalizations with HCC per 1,000,000 of Canadian population, by sex
| Category | Female | Male |
|---|---|---|
| 2006 | 7.609694507292066 | 23.475110405797942 |
| 2007 | 6.931814393978893 | 26.99576632759166 |
| 2008 | 6.617802245760135 | 27.922485722808144 |
| 2009 | 8.723595356736697 | 35.826994145797144 |
| 2010 | 10.374734234862768 | 36.0877485476907 |
| 2011 | 11.082379017362394 | 38.43773979507395 |
| 2012 | 11.71139333474327 | 37.07168548335551 |
| 2013 | 12.215958464383892 | 46.14633560454142 |
| 2014 | 11.032867977789548 | 41.918592954934894 |
| 2015 | 9.616396389415584 | 45.22153215632017 |
| 2016 | 9.61908902170072 | 42.92133908326717 |
| 2017 | 7.116065969299445 | 32.035397956836356 |
| 2018 | 8.414260295410209 | 31.02201400347838 |
| 2019 | 6.713004013794959 | 25.210455112945247 |
