# Supplementary material for: The impact of expanded access to direct acting antivirals for Hepatitis C virus on patient outcomes in Canada
Source: PLoS One. 2023 Aug 8;18(8):e0284914. doi: 10.1371/journal.pone.0284914 (PMC10409286; doi:10.1371/journal.pone.0284914)
Supplement: S5 Fig — (PPTX) [file pone.0284914.s007.pptx]

## Slide 1
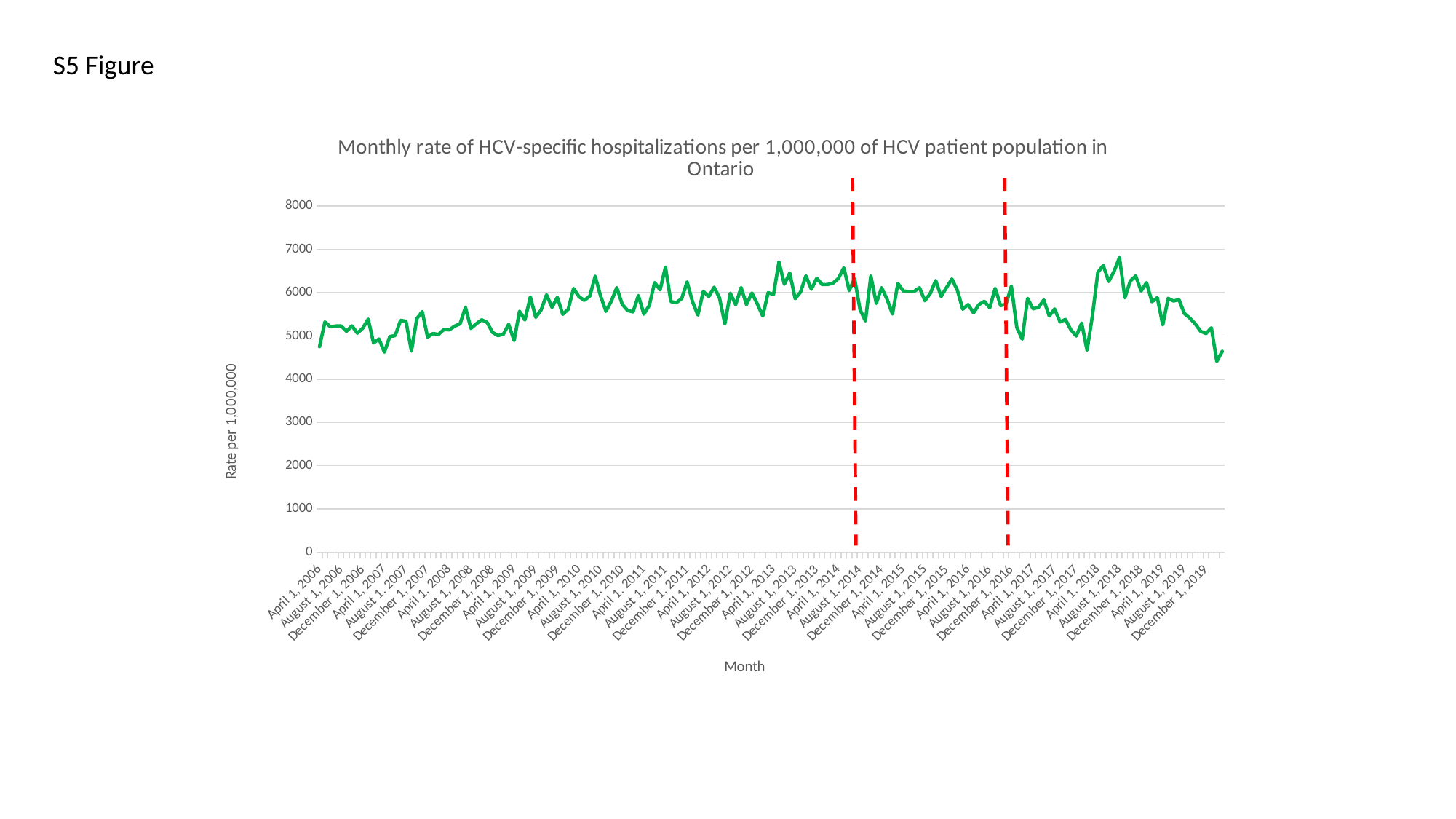

S5 Figure
### Chart: Monthly rate of HCV-specific hospitalizations per 1,000,000 of HCV patient population in Ontario
| Category | Rate of Hosp |
|---|---|
| 38808 | 4751.607273626359 |
| 38838 | 5320.887864958882 |
| 38869 | 5209.373446952357 |
| 38899 | 5227.791529557595 |
| 38930 | 5228.5356574656735 |
| 38961 | 5107.136230884429 |
| 38991 | 5230.02454898127 |
| 39022 | 5062.773801994747 |
| 39052 | 5178.053558742187 |
| 39083 | 5385.026176915871 |
| 39114 | 4835.754872058415 |
| 39142 | 4928.130023660297 |
| 39173 | 4623.168278782226 |
| 39203 | 4983.033597920228 |
| 39234 | 5006.675234793831 |
| 39264 | 5359.052967180073 |
| 39295 | 5336.384318389956 |
| 39326 | 4648.543529752363 |
| 39356 | 5398.084407897301 |
| 39387 | 5558.927747994877 |
| 39417 | 4970.403249871555 |
| 39448 | 5054.7697513014555 |
| 39479 | 5032.079151209034 |
| 39508 | 5147.0488124422445 |
| 39539 | 5139.657149376206 |
| 39569 | 5224.048917554523 |
| 39600 | 5277.85285383584 |
| 39630 | 5660.588229113367 |
| 39661 | 5171.51968789334 |
| 39692 | 5279.128378874239 |
| 39722 | 5371.454421919162 |
| 39753 | 5310.750608267662 |
| 39783 | 5081.666674301072 |
| 39814 | 5005.615719144534 |
| 39845 | 5036.7143047814825 |
| 39873 | 5266.857061371924 |
| 39904 | 4892.216028610377 |
| 39934 | 5566.482615661933 |
| 39965 | 5367.921379635699 |
| 39995 | 5896.856234308044 |
| 40026 | 5429.634293797902 |
| 40057 | 5598.043263072103 |
| 40087 | 5950.239160750088 |
| 40118 | 5659.165190072111 |
| 40148 | 5888.827084850239 |
| 40179 | 5498.211431055709 |
| 40210 | 5613.005873191284 |
| 40238 | 6095.356616547382 |
| 40269 | 5903.845176277654 |
| 40299 | 5819.54062430005 |
| 40330 | 5919.010848394475 |
| 40360 | 6378.362145331861 |
| 40391 | 5919.432064376364 |
| 40422 | 5567.642421086412 |
| 40452 | 5805.538514455468 |
| 40483 | 6112.411440828548 |
| 40513 | 5729.908319532661 |
| 40544 | 5584.830456241785 |
| 40575 | 5554.652114444851 |
| 40603 | 5930.566969271949 |
| 40634 | 5501.9431278828615 |
| 40664 | 5701.655722120951 |
| 40695 | 6230.960601644932 |
| 40725 | 6062.857365872185 |
| 40756 | 6586.627372410173 |
| 40787 | 5793.280526058403 |
| 40817 | 5765.8630194937205 |
| 40848 | 5860.745914049942 |
| 40878 | 6245.621291212119 |
| 40909 | 5790.675004130892 |
| 40940 | 5481.275561876649 |
| 40969 | 6025.523951872868 |
| 41000 | 5906.713416978918 |
| 41030 | 6122.7448473247805 |
| 41061 | 5874.841225053339 |
| 41091 | 5277.983877352523 |
| 41122 | 5985.043991797458 |
| 41153 | 5719.973123388331 |
| 41183 | 6115.793071058072 |
| 41214 | 5721.49834081867 |
| 41244 | 5988.236213309225 |
| 41275 | 5738.22496779845 |
| 41306 | 5457.741783992672 |
| 41334 | 5998.234940684634 |
| 41365 | 5953.415142381398 |
| 41395 | 6707.040597391271 |
| 41426 | 6190.770534923903 |
| 41456 | 6450.213858513884 |
| 41487 | 5858.279895886756 |
| 41518 | 6011.84310108932 |
| 41548 | 6386.217196931337 |
| 41579 | 6075.553040805592 |
| 41609 | 6328.272529727414 |
| 41640 | 6185.024550895102 |
| 41671 | 6186.466989508572 |
| 41699 | 6218.392416843585 |
| 41730 | 6326.556312110439 |
| 41760 | 6572.005139589602 |
| 41791 | 6047.3510815447235 |
| 41821 | 6323.360121105602 |
| 41852 | 5608.288254553502 |
| 41883 | 5343.067770463287 |
| 41913 | 6383.349539119242 |
| 41944 | 5751.57890974443 |
| 41974 | 6112.683401032051 |
| 42005 | 5847.270858213965 |
| 42036 | 5505.213747915881 |
| 42064 | 6210.801983558618 |
| 42095 | 6036.96521255302 |
| 42125 | 6023.743498126965 |
| 42156 | 6025.826049582387 |
| 42186 | 6112.162913755746 |
| 42217 | 5812.801306416909 |
| 42248 | 5980.622820350304 |
| 42278 | 6278.572522571672 |
| 42309 | 5910.388807133926 |
| 42339 | 6116.418932755663 |
| 42370 | 6314.748991586444 |
| 42401 | 6053.831538701909 |
| 42430 | 5616.963707097604 |
| 42461 | 5723.462884761242 |
| 42491 | 5531.555711960669 |
| 42522 | 5722.190569386472 |
| 42552 | 5798.045995776499 |
| 42583 | 5648.632778088834 |
| 42614 | 6096.418197635026 |
| 42644 | 5701.767385458095 |
| 42675 | 5728.3851826031705 |
| 42705 | 6146.379224877572 |
| 42736 | 5198.17510085383 |
| 42767 | 4924.882122021235 |
| 42795 | 5865.946503995803 |
| 42826 | 5623.502100930578 |
| 42856 | 5657.869472081431 |
| 42887 | 5830.9288241720205 |
| 42917 | 5456.970047718058 |
| 42948 | 5621.215256587056 |
| 42979 | 5322.752010900996 |
| 43009 | 5379.0360188339655 |
| 43040 | 5141.981552738159 |
| 43070 | 4997.413147400274 |
| 43101 | 5293.17574450325 |
| 43132 | 4669.2542993705065 |
| 43160 | 5467.829046380575 |
| 43191 | 6468.249972941669 |
| 43221 | 6625.815910789096 |
| 43252 | 6256.941914435525 |
| 43282 | 6492.022582508301 |
| 43313 | 6809.853455091291 |
| 43344 | 5880.355133472943 |
| 43374 | 6267.916318730486 |
| 43405 | 6384.321791636562 |
| 43435 | 6035.842576563123 |
| 43466 | 6229.732303986295 |
| 43497 | 5788.243974979458 |
| 43525 | 5881.402381110936 |
| 43556 | 5253.8987851974725 |
| 43586 | 5866.252860001688 |
| 43617 | 5804.430411689299 |
| 43647 | 5835.601933272144 |
| 43678 | 5518.023801305912 |
| 43709 | 5409.683927901125 |
| 43739 | 5278.086140853725 |
| 43770 | 5107.72692123195 |
| 43800 | 5053.621893024828 |
| 43831 | 5185.5420669193945 |
| 43862 | 4410.554624647323 |
| 43891 | 4643.2349137034225 |
