# Supplementary material for: The impact of expanded access to direct acting antivirals for Hepatitis C virus on patient outcomes in Canada
Source: PLoS One. 2023 Aug 8;18(8):e0284914. doi: 10.1371/journal.pone.0284914 (PMC10409286; doi:10.1371/journal.pone.0284914)
Supplement: S6 Fig — (PPTX) [file pone.0284914.s008.pptx]

## Slide 1
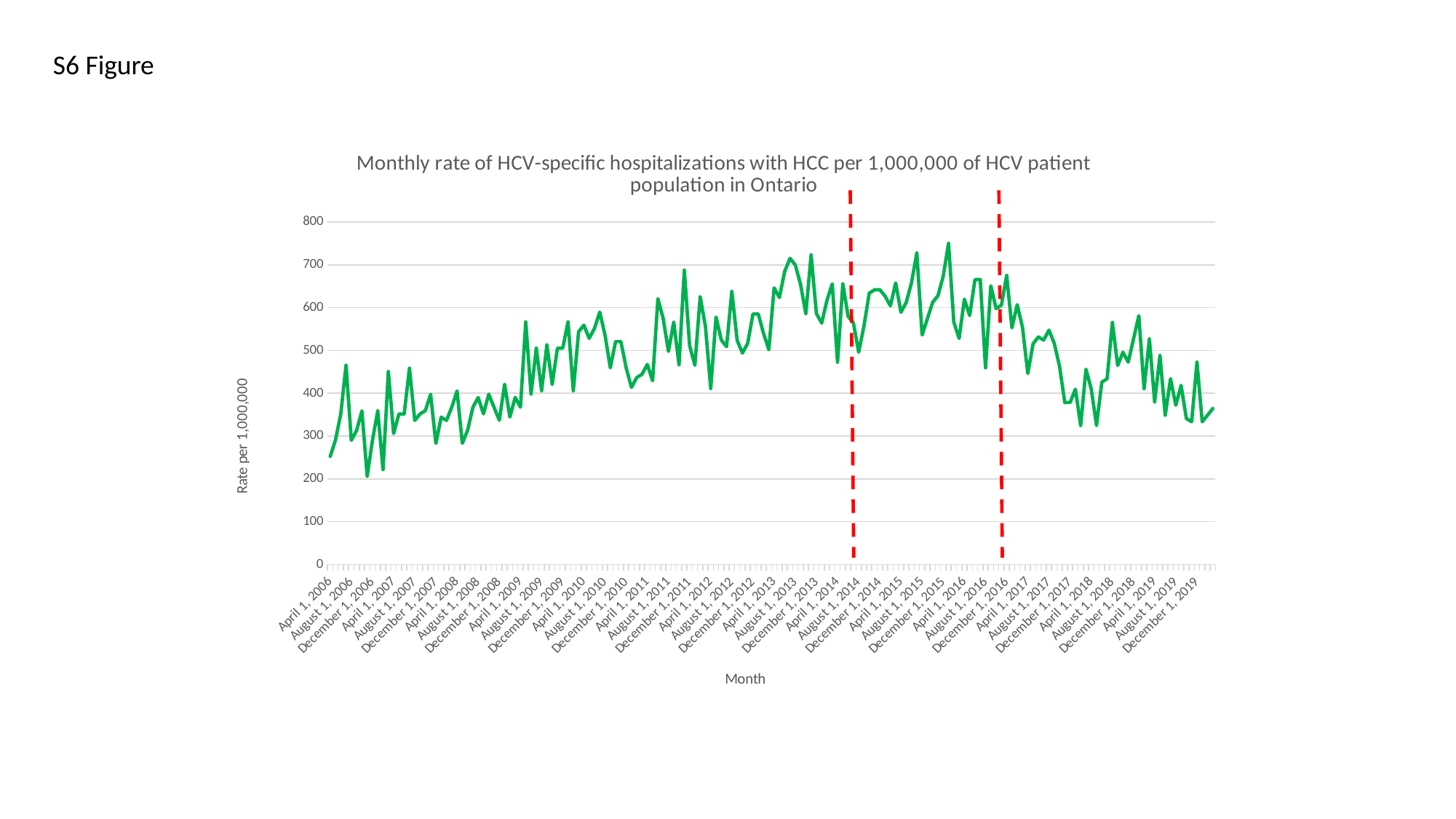

S6 Figure
### Chart: Monthly rate of HCV-specific hospitalizations with HCC per 1,000,000 of HCV patient population in Ontario
| Category | Rate of HCC |
|---|---|
| 38808 | 252.5008696129949 |
| 38838 | 290.5082455006286 |
| 38869 | 351.3653644571971 |
| 38899 | 465.5405595664427 |
| 38930 | 290.0501532608695 |
| 38961 | 312.99340129486035 |
| 38991 | 358.84839971112365 |
| 39022 | 206.17630867851912 |
| 39052 | 290.2153911979397 |
| 39083 | 359.0017451277247 |
| 39114 | 221.5432721796114 |
| 39142 | 450.7901882107868 |
| 39173 | 305.66401843188265 |
| 39203 | 351.5637200986664 |
| 39234 | 351.61383328323086 |
| 39264 | 458.69212272582655 |
| 39295 | 336.3909885518024 |
| 39326 | 351.69901705363264 |
| 39356 | 359.36255973253986 |
| 39387 | 397.61243864612595 |
| 39417 | 282.93064653115005 |
| 39448 | 344.12199517180863 |
| 39479 | 336.49161497446426 |
| 39508 | 367.10006388889707 |
| 39539 | 405.3598644597305 |
| 39569 | 283.00118587044994 |
| 39600 | 313.611546387347 |
| 39630 | 367.17329053708323 |
| 39661 | 390.1590297079295 |
| 39692 | 351.9418919249493 |
| 39722 | 397.88551273475275 |
| 39753 | 367.3141631078499 |
| 39783 | 336.736948297059 |
| 39814 | 420.9615665947238 |
| 39845 | 344.4561454637792 |
| 39873 | 390.42109030518617 |
| 39904 | 367.49040590500493 |
| 39934 | 566.6020819243233 |
| 39965 | 398.1910295878122 |
| 39995 | 505.44482008354674 |
| 40026 | 405.8823943177557 |
| 40057 | 513.090148598948 |
| 40087 | 421.18810018179505 |
| 40118 | 505.4193539171303 |
| 40148 | 505.4129877764834 |
| 40179 | 566.6680304987777 |
| 40210 | 405.85172070823745 |
| 40238 | 543.6813062498293 |
| 40269 | 558.9892319951605 |
| 40299 | 528.3530303640833 |
| 40330 | 551.3179574183729 |
| 40360 | 589.5965008289957 |
| 40391 | 536.041713462284 |
| 40422 | 459.50281329461444 |
| 40452 | 520.8134815078785 |
| 40483 | 520.857115258573 |
| 40513 | 459.6183144010156 |
| 40544 | 413.6911449067989 |
| 40575 | 436.71058003221583 |
| 40603 | 444.4094111340737 |
| 40634 | 467.43527966692835 |
| 40664 | 429.1568823101791 |
| 40695 | 620.7968127100116 |
| 40725 | 574.8600536541262 |
| 40756 | 497.82648744960613 |
| 40787 | 566.3180434984436 |
| 40817 | 466.46902412349726 |
| 40848 | 687.7016066029919 |
| 40878 | 511.5606681066161 |
| 40909 | 465.3902177232996 |
| 40940 | 625.1246120638181 |
| 40969 | 556.0850170502141 |
| 41000 | 411.03418107842987 |
| 41030 | 578.0479607412216 |
| 41061 | 524.4036798559902 |
| 41091 | 508.81283421959574 |
| 41122 | 637.9996133388154 |
| 41153 | 524.1409634977356 |
| 41183 | 493.8218007686642 |
| 41214 | 516.6824530885385 |
| 41244 | 585.1449091685411 |
| 41275 | 585.222943735736 |
| 41306 | 539.6931290577712 |
| 41334 | 501.7534931371177 |
| 41365 | 646.2838915739704 |
| 41395 | 623.5570623424991 |
| 41426 | 684.48322867709 |
| 41456 | 715.0001211088503 |
| 41487 | 699.9503252228333 |
| 41518 | 654.4538059413692 |
| 41548 | 586.1009823167019 |
| 41579 | 723.2801239054276 |
| 41609 | 586.3742295896641 |
| 41640 | 563.6598728648246 |
| 41671 | 617.1229386086136 |
| 41699 | 655.3697890300838 |
| 41730 | 472.5861341576472 |
| 41760 | 655.6756867804011 |
| 41791 | 579.5695866297591 |
| 41821 | 564.4495162386183 |
| 41852 | 495.9710701305818 |
| 41883 | 557.2056389197428 |
| 41913 | 633.7536025680587 |
| 41944 | 641.6103963061515 |
| 41974 | 641.8317571083654 |
| 42005 | 626.7662880699936 |
| 42036 | 604.0442862296592 |
| 42064 | 657.7942987512822 |
| 42095 | 589.1588356990908 |
| 42125 | 612.3246249684336 |
| 42156 | 658.4765441729164 |
| 42186 | 727.6384421137792 |
| 42217 | 536.0949821464869 |
| 42248 | 574.3235742974043 |
| 42278 | 612.5436607386997 |
| 42309 | 627.7874121567124 |
| 42339 | 673.6481427815999 |
| 42370 | 750.1156377884503 |
| 42401 | 566.3508645561836 |
| 42430 | 528.0251986236168 |
| 42461 | 619.7867562375142 |
| 42491 | 581.46367096682 |
| 42522 | 665.5489031238276 |
| 42552 | 665.4749361907063 |
| 42583 | 459.2384372429947 |
| 42614 | 650.9994306519815 |
| 42644 | 597.7659355722196 |
| 42675 | 605.8131585350072 |
| 42705 | 675.2576426831789 |
| 42736 | 552.8339841380737 |
| 42767 | 606.9667513879524 |
| 42795 | 553.5362362879395 |
| 42826 | 446.1875811955862 |
| 42856 | 515.7513668427971 |
| 42887 | 531.484925849233 |
| 42917 | 524.1157672949547 |
| 42948 | 547.4708960462016 |
| 42979 | 516.8469343918358 |
| 43009 | 463.04470750364123 |
| 43040 | 378.313958084339 |
| 43070 | 378.47487515087084 |
| 43101 | 409.54498461120033 |
| 43132 | 324.6832459827173 |
| 43160 | 456.29690768946807 |
| 43191 | 410.06847914582346 |
| 43221 | 325.09844422096035 |
| 43252 | 425.90569962123 |
| 43282 | 433.8344446544926 |
| 43313 | 565.5509695354542 |
| 43344 | 464.8502081796793 |
| 43374 | 495.8549374521027 |
| 43405 | 472.6257637012504 |
| 43435 | 526.8771440388861 |
| 43466 | 581.131744774841 |
| 43497 | 410.67862205342874 |
| 43525 | 526.9240604947875 |
| 43556 | 379.70654937267875 |
| 43586 | 488.20862639379965 |
| 43617 | 348.73079910015815 |
| 43647 | 433.98898839739724 |
| 43678 | 372.001604582421 |
| 43709 | 418.51422937917016 |
| 43739 | 341.0217183517825 |
| 43770 | 333.28111929131086 |
| 43800 | 472.8081832431205 |
| 43831 | 333.300910130843 |
| 43862 | 348.813634638189 |
| 43891 | 364.3272803740582 |
