# Supplementary material for: The impact of expanded access to direct acting antivirals for Hepatitis C virus on patient outcomes in Canada
Source: PLoS One. 2023 Aug 8;18(8):e0284914. doi: 10.1371/journal.pone.0284914 (PMC10409286; doi:10.1371/journal.pone.0284914)
Supplement: S7 Fig — (PPTX) [file pone.0284914.s009.pptx]

## Slide 1
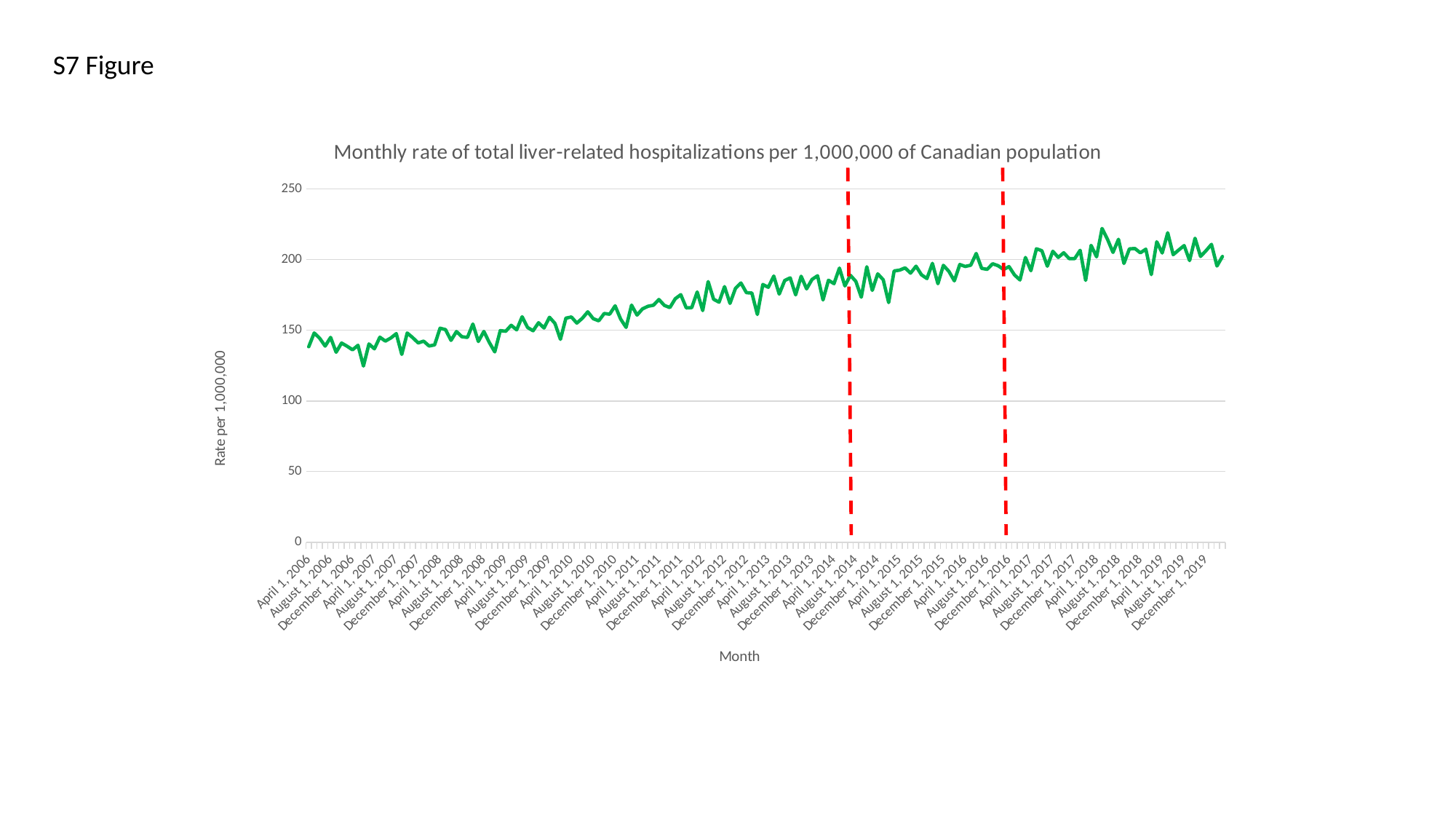

S7 Figure
### Chart: Monthly rate of total liver-related hospitalizations per 1,000,000 of Canadian population
| Category | Hosp rate per 1M |
|---|---|
| 38808 | 138.37259233460185 |
| 38838 | 148.1051827789422 |
| 38869 | 144.3255546865539 |
| 38899 | 138.80371643957324 |
| 38930 | 144.96581889791628 |
| 38961 | 134.44961400901465 |
| 38991 | 140.97000089838926 |
| 39022 | 138.7451203301416 |
| 39052 | 136.15500507898224 |
| 39083 | 139.37409599775972 |
| 39114 | 124.70803719628721 |
| 39142 | 140.3401977026511 |
| 39173 | 136.85713670469366 |
| 39203 | 145.12337177624946 |
| 39234 | 142.3541139699759 |
| 39264 | 144.54669908882977 |
| 39295 | 147.63081843901733 |
| 39326 | 132.9008119569191 |
| 39356 | 148.0813152166081 |
| 39387 | 144.79850661384316 |
| 39417 | 141.0345666974534 |
| 39448 | 142.32716349315518 |
| 39479 | 138.89120396623775 |
| 39508 | 139.65972309753604 |
| 39539 | 151.475268900298 |
| 39569 | 150.4791484019874 |
| 39600 | 142.86036074288833 |
| 39630 | 149.09562988286683 |
| 39661 | 145.42391697633127 |
| 39692 | 144.97166442011925 |
| 39722 | 154.43891419554913 |
| 39753 | 142.0757916048421 |
| 39783 | 149.1521435474791 |
| 39814 | 141.38250309255528 |
| 39845 | 134.71777492985007 |
| 39873 | 149.7766043866775 |
| 39904 | 149.32287715104843 |
| 39934 | 153.55964338117548 |
| 39965 | 150.31398773026226 |
| 39995 | 159.56515966403296 |
| 40026 | 151.96772966102105 |
| 40057 | 149.66920761900306 |
| 40087 | 155.34520361463763 |
| 40118 | 151.6259851309418 |
| 40148 | 159.21535045108794 |
| 40179 | 154.8471643698065 |
| 40210 | 143.580132678919 |
| 40238 | 158.58484280722897 |
| 40269 | 159.43115389205 |
| 40299 | 155.09411209166103 |
| 40330 | 158.50987527710524 |
| 40360 | 163.0647875368745 |
| 40391 | 158.1923720476252 |
| 40422 | 156.70551922490517 |
| 40452 | 161.87306087426163 |
| 40483 | 161.40863373639604 |
| 40513 | 167.32725554285807 |
| 40544 | 157.96360011718048 |
| 40575 | 152.04376301936878 |
| 40603 | 167.8215706795263 |
| 40634 | 160.7636351547155 |
| 40664 | 165.17496230839657 |
| 40695 | 166.98242449734784 |
| 40725 | 167.76682409160716 |
| 40756 | 171.76240757238452 |
| 40787 | 167.6136331720774 |
| 40817 | 166.08625956737242 |
| 40848 | 172.3735402390749 |
| 40878 | 175.2041453926943 |
| 40909 | 165.83444275918845 |
| 40940 | 166.03058921327295 |
| 40969 | 177.1327066203506 |
| 41000 | 163.96485520865497 |
| 41030 | 184.4758477833158 |
| 41061 | 171.94568073863158 |
| 41091 | 169.8727397664277 |
| 41122 | 180.89578603453455 |
| 41153 | 169.04527204055384 |
| 41183 | 179.6991205192053 |
| 41214 | 183.49170511807 |
| 41244 | 176.6405665897461 |
| 41275 | 176.3317093290884 |
| 41306 | 161.19444270389798 |
| 41334 | 182.40536164129173 |
| 41365 | 180.44399062011632 |
| 41395 | 188.40110169787087 |
| 41426 | 175.62096403295592 |
| 41456 | 185.24665853394217 |
| 41487 | 187.12674648071427 |
| 41518 | 175.0979080131472 |
| 41548 | 188.23200111008032 |
| 41579 | 179.30723531096592 |
| 41609 | 186.02579875590033 |
| 41640 | 188.59767613506625 |
| 41671 | 171.4847661720118 |
| 41699 | 185.44486346388206 |
| 41730 | 182.99415293481545 |
| 41760 | 194.0245729204118 |
| 41791 | 181.38690842648543 |
| 41821 | 188.95272753233976 |
| 41852 | 184.62159952264088 |
| 41883 | 173.40254582190659 |
| 41913 | 194.97147941000782 |
| 41944 | 178.2399024733338 |
| 41974 | 189.93636934830712 |
| 42005 | 185.77555193174388 |
| 42036 | 169.61147423309185 |
| 42064 | 192.02655517141193 |
| 42095 | 192.57947855890907 |
| 42125 | 194.1820212119753 |
| 42156 | 190.4555671562991 |
| 42186 | 195.36223772024397 |
| 42217 | 189.24036295876354 |
| 42248 | 186.62546212950735 |
| 42278 | 197.35982246437152 |
| 42309 | 182.98514566878794 |
| 42339 | 195.95563121243177 |
| 42370 | 191.76978392878328 |
| 42401 | 185.02017986293617 |
| 42430 | 196.5648602542755 |
| 42461 | 195.18879226566426 |
| 42491 | 196.04795712670048 |
| 42522 | 204.36406095957184 |
| 42552 | 193.82717899038553 |
| 42583 | 193.14568800776564 |
| 42614 | 197.1058026407243 |
| 42644 | 195.7321942309832 |
| 42675 | 193.04144622546087 |
| 42705 | 195.0922959307768 |
| 42736 | 189.18271936197564 |
| 42767 | 185.62508789744146 |
| 42795 | 201.53949965688332 |
| 42826 | 192.15334639283742 |
| 42856 | 207.67091566951183 |
| 42887 | 206.35046690069734 |
| 42917 | 195.3743026861285 |
| 42948 | 205.90703021203706 |
| 42979 | 201.59530228121363 |
| 43009 | 204.89513652636242 |
| 43040 | 200.72413444837792 |
| 43070 | 200.74686062902185 |
| 43101 | 206.61219909296076 |
| 43132 | 185.30208815226231 |
| 43160 | 210.09035919890607 |
| 43191 | 201.98690833222693 |
| 43221 | 222.08702704893886 |
| 43252 | 214.37719843695407 |
| 43282 | 205.12566489799403 |
| 43313 | 214.40008472574203 |
| 43344 | 197.31843759292158 |
| 43374 | 207.6220151818565 |
| 43405 | 207.91228301502858 |
| 43435 | 204.82417225498412 |
| 43466 | 207.41999347090515 |
| 43497 | 189.4115550114043 |
| 43525 | 212.64830476384532 |
| 43556 | 204.7406957218173 |
| 43586 | 219.03628622882852 |
| 43617 | 203.50648058929082 |
| 43647 | 206.8017455811951 |
| 43678 | 209.9767761969123 |
| 43709 | 199.3352735217522 |
| 43739 | 215.12805673456344 |
| 43770 | 202.25655494406953 |
| 43800 | 206.33111334139008 |
| 43831 | 210.8206438887693 |
| 43862 | 195.49369116341103 |
| 43891 | 202.23677508607312 |
