# Supplementary material for: The impact of expanded access to direct acting antivirals for Hepatitis C virus on patient outcomes in Canada
Source: PLoS One. 2023 Aug 8;18(8):e0284914. doi: 10.1371/journal.pone.0284914 (PMC10409286; doi:10.1371/journal.pone.0284914)
Supplement: S8 Fig — (PPTX) [file pone.0284914.s010.pptx]

## Slide 1
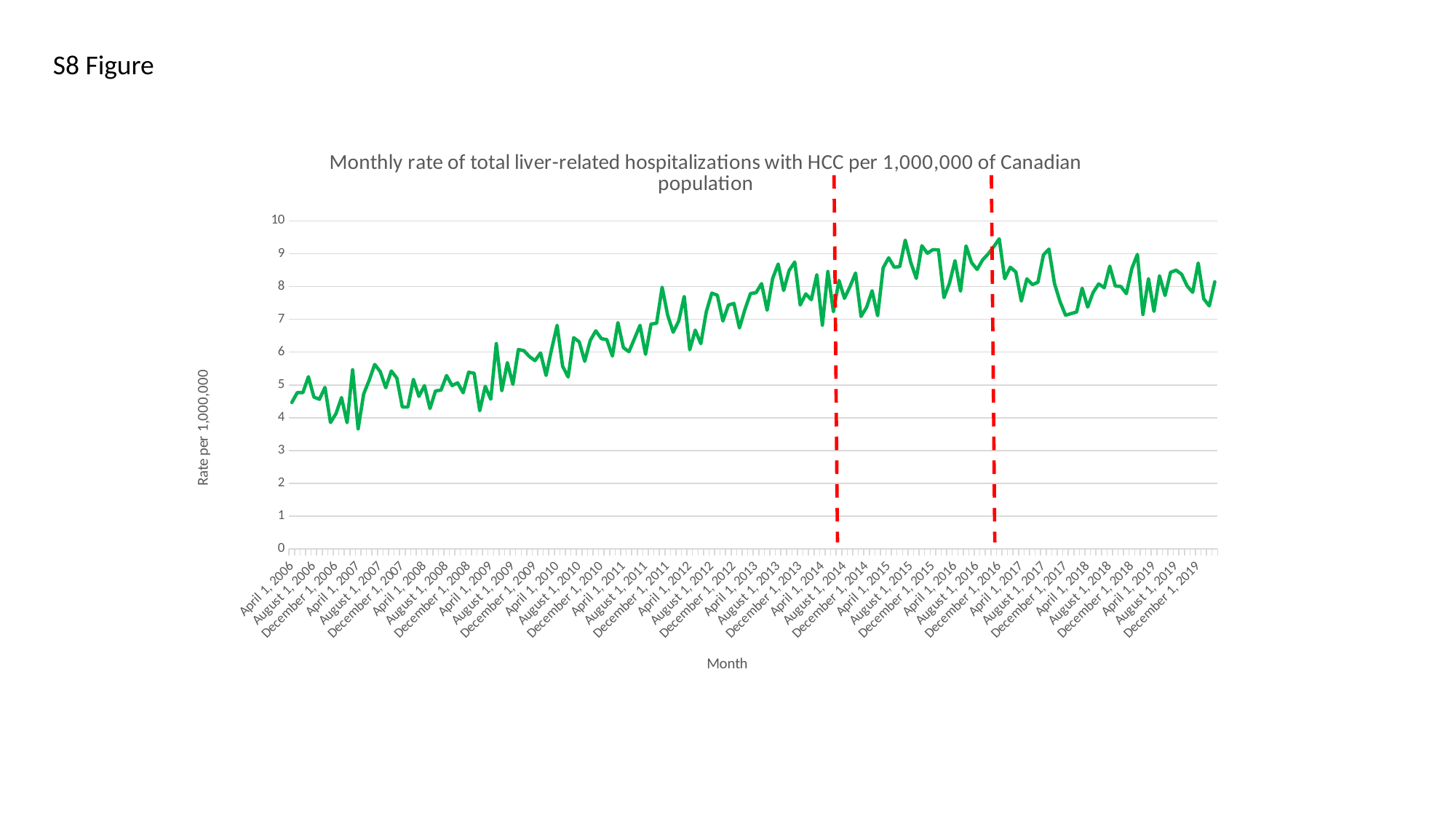

S8 Figure
### Chart: Monthly rate of total liver-related hospitalizations with HCC per 1,000,000 of Canadian population
| Category | HCC rate per 1M |
|---|---|
| 38808 | 4.465618938018533 |
| 38838 | 4.768654617934366 |
| 38869 | 4.763726783734211 |
| 38899 | 5.250041033215443 |
| 38930 | 4.630809954217338 |
| 38961 | 4.5643637474010434 |
| 38991 | 4.92645325475161 |
| 39022 | 3.8540311202817112 |
| 39052 | 4.127762336775792 |
| 39083 | 4.6152387051889745 |
| 39114 | 3.8484478782101856 |
| 39142 | 5.463439623482937 |
| 39173 | 3.660097259764484 |
| 39203 | 4.722679535023864 |
| 39234 | 5.14386257449774 |
| 39264 | 5.624976720957827 |
| 39295 | 5.4059423332946075 |
| 39326 | 4.9143874770648015 |
| 39356 | 5.423891021848342 |
| 39387 | 5.209233034423975 |
| 39417 | 4.3288137020252915 |
| 39448 | 4.326697359592091 |
| 39479 | 5.169872851159481 |
| 39508 | 4.652303127194582 |
| 39539 | 4.980753162325462 |
| 39569 | 4.281314180140695 |
| 39600 | 4.818224645628612 |
| 39630 | 4.842524997204269 |
| 39661 | 5.287050069786057 |
| 39692 | 4.980400723042176 |
| 39722 | 5.064062184526156 |
| 39753 | 4.761815106486065 |
| 39783 | 5.38779567299744 |
| 39814 | 5.354944573332077 |
| 39845 | 4.214600901954484 |
| 39873 | 4.957710135231998 |
| 39904 | 4.565627538791049 |
| 39934 | 6.259224594341393 |
| 39965 | 4.822908697227666 |
| 39995 | 5.679639488600503 |
| 40026 | 5.019063184036068 |
| 40057 | 6.080496940526283 |
| 40087 | 6.043177257319998 |
| 40118 | 5.862516121055746 |
| 40148 | 5.7412226742585615 |
| 40179 | 5.975000420764262 |
| 40210 | 5.290416580800021 |
| 40238 | 6.083515385156269 |
| 40269 | 6.816323625590143 |
| 40299 | 5.570655109335601 |
| 40330 | 5.2404825035892895 |
| 40360 | 6.440250400464475 |
| 40391 | 6.314771628340034 |
| 40422 | 5.720250140182797 |
| 40452 | 6.357729268726655 |
| 40483 | 6.648477564536728 |
| 40513 | 6.412015566734194 |
| 40544 | 6.38059381610994 |
| 40575 | 5.87933750805947 |
| 40603 | 6.898779076880023 |
| 40634 | 6.134901577773988 |
| 40664 | 6.011668239492878 |
| 40695 | 6.413431108487521 |
| 40725 | 6.814344182856461 |
| 40756 | 5.9338748763364 |
| 40787 | 6.856789292530815 |
| 40817 | 6.877938758949374 |
| 40848 | 7.976228094522227 |
| 40878 | 7.131075408107677 |
| 40909 | 6.605564805921553 |
| 40940 | 6.948097892098607 |
| 40969 | 7.6951330983199835 |
| 41000 | 6.070631099051048 |
| 41030 | 6.669889002652363 |
| 41061 | 6.258338262375554 |
| 41091 | 7.230465945628855 |
| 41122 | 7.7974801996753405 |
| 41153 | 7.730904144663207 |
| 41183 | 6.946835010486851 |
| 41214 | 7.431485789770154 |
| 41244 | 7.4854989251378035 |
| 41275 | 6.736782912101409 |
| 41306 | 7.3048841104485485 |
| 41334 | 7.786292901197638 |
| 41365 | 7.809322992912453 |
| 41395 | 8.085761568167646 |
| 41426 | 7.277111769321378 |
| 41456 | 8.237618759241311 |
| 41487 | 8.683045439923603 |
| 41518 | 7.876278096726496 |
| 41548 | 8.491455692805374 |
| 41579 | 8.743924711174397 |
| 41609 | 7.43535610588038 |
| 41640 | 7.773129251054173 |
| 41671 | 7.597605775185842 |
| 41699 | 8.357200538014851 |
| 41730 | 6.822647100447172 |
| 41760 | 8.455523583035 |
| 41791 | 7.231747166668786 |
| 41821 | 8.18343652693825 |
| 41852 | 7.638542514600867 |
| 41883 | 7.995830981234206 |
| 41913 | 8.408549306734797 |
| 41944 | 7.085732043426427 |
| 41974 | 7.365797627184201 |
| 42005 | 7.87065434118449 |
| 42036 | 7.109294728457959 |
| 42064 | 8.567597912124143 |
| 42095 | 8.873595104201701 |
| 42125 | 8.585420963858464 |
| 42156 | 8.606102313362353 |
| 42186 | 9.410998118136483 |
| 42217 | 8.729005506081347 |
| 42248 | 8.244161624469102 |
| 42278 | 9.239901164880761 |
| 42309 | 9.012534621991232 |
| 42339 | 9.120052861722913 |
| 42370 | 9.115964434468985 |
| 42401 | 7.659272837920736 |
| 42430 | 8.097448235276637 |
| 42461 | 8.785024691062514 |
| 42491 | 7.857466628927382 |
| 42522 | 9.233817136979297 |
| 42552 | 8.723469264462272 |
| 42583 | 8.517879711682678 |
| 42614 | 8.809969320777785 |
| 42644 | 8.99093917420044 |
| 42675 | 9.206888910367546 |
| 42705 | 9.450170527362864 |
| 42736 | 8.233716606874921 |
| 42767 | 8.585091524459196 |
| 42795 | 8.44101315070439 |
| 42826 | 7.555357486135301 |
| 42856 | 8.23111041099928 |
| 42887 | 8.055641650352547 |
| 42917 | 8.126914271397782 |
| 42948 | 8.960794203203948 |
| 42979 | 9.137386857557376 |
| 43009 | 8.087965915514307 |
| 43040 | 7.538040298562999 |
| 43070 | 7.124854712111044 |
| 43101 | 7.174223406621286 |
| 43132 | 7.222030102344583 |
| 43160 | 7.947898675956033 |
| 43191 | 7.371587155020223 |
| 43221 | 7.820537445734536 |
| 43252 | 8.078999537767743 |
| 43282 | 7.958972924491416 |
| 43313 | 8.619098883446915 |
| 43344 | 8.013204470249473 |
| 43374 | 7.999917316290825 |
| 43405 | 7.779943493465585 |
| 43435 | 8.552213475044493 |
| 43466 | 8.975161174470838 |
| 43497 | 7.147100789718054 |
| 43525 | 8.237413893505765 |
| 43556 | 7.2415464031078685 |
| 43586 | 8.323912460827588 |
| 43617 | 7.7247224307453335 |
| 43647 | 8.430575276393885 |
| 43678 | 8.49463569949582 |
| 43709 | 8.372982378422662 |
| 43739 | 8.013744921388337 |
| 43770 | 7.821784722164173 |
| 43800 | 8.712638183321653 |
| 43831 | 7.623519279761553 |
| 43862 | 7.407460520080702 |
| 43891 | 8.140049954617245 |
